# Manipulation of Plant Defense Responses by the Tomato Psyllid (*Bactericerca cockerelli*) and Its Associated Endosymbiont *Candidatus* Liberibacter Psyllaurous

**DOI:** 10.1371/journal.pone.0035191

**Published:** 2012-04-23

**Authors:** Clare L. Casteel, Allison K. Hansen, Linda L. Walling, Timothy D. Paine

**Affiliations:** 1 Boyce Thompson Institute for Plant Research, Ithaca, New York, United States of America; 2 Department of Ecology and Evolutionary Biology, Yale, New Haven, Connecticut, United States of America; 3 Department of Botany and Plant Sciences, University of California Riverside, Riverside, California, United States of America; 4 Department of Entomology, University of California Riverside, Riverside, California, United States of America; 5 Center for Disease-Vector Research, University of California Riverside, Riverside, California, United States of America; Max Planck Institute for Chemical Ecology, Germany

## Abstract

Some plant pathogens form obligate relationships with their insect vector and are vertically transmitted via eggs analogous to insect endosymbionts. Whether insect endosymbionts manipulate plant defenses to benefit their insect host remains unclear. The tomato psyllid, *Bactericerca cockerelli* (Sulc), vectors the endosymbiont “*Candidatus* Liberibacter psyllaurous” *(Lps)* during feeding on tomato (*Solanum lycopersicum* L.). *Lps* titer in psyllids varied relative to the psyllid developmental stage with younger psyllids harboring smaller *Lps* populations compared to older psyllids. In the present study, feeding by different life stages of *B. cockerelli* infected with *Lps*, resulted in distinct tomato transcript profiles. Feeding by young psyllid nymphs, with lower *Lps* levels, induced tomato genes regulated by jasmonic acid (JA) and salicylic acid (SA) (*Allene oxide synthase*, *Proteinase inhibitor 2*, *Phenylalanine ammonia-lyase 5*, *Pathogenesis-related protein 1*) compared to feeding by older nymphs and adults, where higher *Lps* titers were found. In addition, inoculation of *Lps* without insect hosts suppressed accumulation of these defense transcripts. Collectively, these data suggest that the endosymbiont-like pathogen *Lps* manipulates plant signaling and defensive responses to benefit themselves and the success of their obligate insect vector on their host plant.

## Introduction

Most bacterial plant pathogens are not vectored by insects [1], [2]. However, the few that are, require insect partners as intermediate hosts [1]. The vast majority of gram-negative bacterial plant pathogens appear to be dependent primarily on one insect vector, are vertically transmitted from parent to offspring, and are confined only to plant tissue in which their insect host feeds [1], [3]–[8]. Given that these pathogens have an obligate relationship with a specific insect vector and are vertically transmitted, these bacteria could be perceived as both an insect endosymbiont and a plant pathogen. To date, the impact of these gram-negative endosymbiont-like plant pathogens on the insect vector-plant interaction has not been studied.

Plants protect themselves against the diversity of herbivores and microbial pathogens by expressing an array of constitutive and induced defenses rendering the plant an inaccessible or unsuitable food source. The perception of attack and deployment of the induced defenses is primarily mediated by three well-studied defense-signaling pathways that are regulated by jasmonic acid (JA), salicylic acid (SA) and ethylene (ET) [9]–[11]. Herbivores and pathogens introduce a distinct set of elicitors and effectors that are perceived by host plants and these signals allow the plant to tailor its defense response to individual challengers [9]–[14].

The SA-regulated defense pathway is activated by biotrophic pathogens (pathogens that invade living plant tissue) and many phloem-feeding insects [9], [11], [15]–[18]. Often SA-induced signaling antagonizes JA- and ET-regulated signaling pathways, although exceptions do exist [19]–[21]. The suppression of JA/ET-regulated defenses confers susceptibility to many tissue-damaging and phloem-feeding herbivores [10], [18], [22], [23] and can influence attraction of natural enemies [24]. However, in some plant-herbivore interactions, SA-regulated defenses and/or novel defense-signaling pathways contribute to the plant immune response [25]–[27].

Therefore, the nature of defenses elicited by endosymbiont-like pathogens in their host plants have the potential to profoundly impact the plant's interaction with the insect and/or ability to resist attacks by other pathogens or pests. If an herbivore can circumvent induced plant defenses or plant recognition by vectoring its endosymbiont associate into its host plant during feeding, it may have a selective advantage relative to insects feeding on uninfected plants. Alternatively, effectors from the endosymbiont may circumvent the plant recognition system, compromising plant immune responses and related insect and bacterial resistance in both the JA/ET- and SA-regulated defense pathways. Thus, the endosymbiont's modification of plant defenses could result in a more susceptible host plant for both symbiotic partners.

A good system to explore insect-endosymbiont elicited plant defense responses is the hemipteran tomato psyllid [*Bactericerca cockerelli* (Sulc)] and its gram-negative endosymbiont “*Candidatus* Liberibacter psyllaurous” [8] (*Lps*) (also known as “*Candidatus* Liberibacter solanacearum” [28]) on tomato (*Solanum lycopersicum* L.). *B. cockerelli* is native to western North America, polyphagous, and can successfully reproduce on a wide variety of plant species [29]–[32]. The insect has been recognized as a major pest of potato (*Solanum tuberosum* L.) and tomato crops for many years [30]–[32]. *B. cockerelli* feeding has been associated with a debilitating plant condition in tomato and potato called “psyllid yellows” [30], [33]. Psyllid yellows disease is now recognized to be associated with *Lps* infection, which is vectored and vertically transmitted by *B. cockerelli* [8].

This study investigates whether the psyllid influences plant defense responses by vectoring *Lps* into tomato plants during feeding. This was first investigated by determining the genome-wide changes in transcript abundance of tomato in response to *Lps*-infected *B. cockerelli* adults and instars. Second, to identify the effect of *Lps* on induced plant defenses, changes in levels of tomato defense transcripts regulated by the JA/ET and SA were determined after feeding by *Lps*-infected *B. cockerelli* and *Lps* infection in the absence of its vector. During plant defense trials we also measured *Lps* infection frequency and titer among different psyllid lifestages and frequency of vertical transmission via eggs to more fully characterize the microbe-insect-plant interaction.

## Results

### Genome-wide transcript profiles of tomato after continuous feeding by developing *Lps*-infected psyllids

To understand the changes in tomato mRNA profiles after *Lps*-infected *B. cockerelli* infestation, potato cDNA arrays were used in a reference design strategy [37]. Potato arrays were used because gene content, genome organization and nucleotide sequence conservation are similar among Solanaceous plant genomes [34].

Arrays were hybridized to cDNAs from tomato leaves after continuous feeding by 1^st^ and 2^nd^ instars (10 days), 3^rd^, 4^th^ and 5^th^ instars (15 days), adult psyllids/egg deposition (2 days), and a no-psyllid control. Consistent with microarray data generated from aphid-infested plants [35], [36], large changes in transcript abundance after psyllid infestation were not found and variation was high, resulting in no statistical differences in transcript levels among treatments (criteria: *P*<0.05 and a fold change of greater than 2). Despite the lack of significant differences, the top 149 expressed transcripts in each treatment are presented in Tables S1, S2, and S3. Non-synchronous development of psyllid infestations may have contributed to variation. For example, although each time point was dominated by insects at a particular stage in development, each infestation actually included eggs, developing instars as well as feeding adults. These mixed, infestations may have induced high variability due to cumulative life-stage effects on tomato mRNAs making life-stage specific effects difficult to resolve.

In order to examine qualitative transcript changes from our microarray data our statistical criteria was relaxed (*P*<0.1 and no fold change limit). Significant differences in 24 transcripts were evident at a *P*<0.1. The greatest numbers of significant transcripts were regulated by 3^rd^–5^th^ instar feeding. Only 5 transcripts were up-regulated significantly (P<0.1), with putative functions in signaling, cell wall synthesis and protein synthesis (Table 1), while 9 transcripts were down-regulated, most with unknown function (Table 1). Feeding by 1^st^–2^nd^ instars down-regulated 2 transcripts significantly, a hypothetical and an oligosaccharide processing protein (Table 1). The largest changes in transcript abundance were after adult feeding/egg deposition. Adults down-regulated transcripts related to signaling, cell wall synthesis/cell division and up-regulated mRNAs related to post-translational regulation (Table 1). Since microarray data could only be interpreted qualitatively the general trend of differential plant expression due to psyllid lifestage helped guide our sample design in the experiments below; the effect of discrete psyllid lifestages on tomato expression.

**Table 1:** Microarray results of differentially expressed transcripts after three developmental stage treatments of psyllids feeding on tomato.

Clone name	TIGR putative function/homology	Ratio of signal intensity (Log2) relative to control		
		Adult/egg deposition	1^st^–2^nd^ instar	3^rd^–5^th^ instar
STMGZ60	Ethylene receptor homolog	−1.4300**	−0.0914	0.0045
STMJA65	similar to RRM-containing protein	−2.0056**	0.0106	0.082
STMDP28	transcription factor D11	0.9470**	0.4574	0.39408
STMJL48	At2g01910 microtubule binding protein	−4.1262**	0.4472	−1.23
STMBB56	Hypothetical protein (serine/theonine kinase)	−0.6323	−1.6648**	−0.1061
STMIN67	Beta-1 2-N-acetylglucosaminyltransferase II	−0.0519	−1.9330**	0.6157
STMCG17	Putative response regulator	−0.7667	−0.4965	−1.9230**
STMCB56	60 S ribosomal protein L10a-1	−0.3779	0.1436	−0.9558**
STMDB96	unknown protein F2E2.11	−0.5736	−0.209	−1.4287**
STMJB40	Ferrous iron transporter (GTP-binding)	0.02099	−0.0033	1.1199**
STMCS61	GTP-binding protein ERG.	−0.3802	−0.3297	−0.9602**
STMCN64	Putative anion transporter	0.029	0.039	−1.2094**
STMCU90	unknown protein F17L21.22	−1.0504	−0.4419	−1.1645**
STMHR03	Diacylglycerol kinase-like protein	0.1309	−0.1352	0.8771**
STMCF01	At2g40480 unknown protein	−0.1228	−0.0395	−1.6650**
STMIQ27	Glycosyltransferase QUASIMODO1	0.0575	0.8629	2.3746**
STMIX47	UDP-glucose 4-epimerase	−0.3431	−0.1461	0.8965**
STMCR29	Ubiquitin conjugating enzyme 2	0.305	0.1514	−1.3282**
STMIX96	unknown protein	−0.0021	0.229	−1.0580**
STMCI66	40 S ribosomal protein SA (p40). {Daucus carota;}	−0.6701	0.2161	2.2762**

** Indicates ratios that are significant to a P value<0.1.

### Tomato responses to graft-transmitted *Lps*

To monitor defense-response gene mRNAs among different psyllid lifestages qRT-PCR was performed. By using primers specific to tomato defense genes, more subtle changes in mRNA levels that were not detected using the heterologous potato arrays and the continuous feeding-design (above) were revealed. To this end, we monitored the defense gene transcripts encoding allene oxide synthase (*AOS*), proteinase inhibitor 2 (*Pin2*), phenylalanine lyase 5 (*PAL5*), and pathogenesis-related 1 (*PR1*)]. *AOS* is important for the synthesis of the defense hormone JA and *Pin2*, a defense transcript in the JA pathway, is known to inhibit Ser proteases critical for digestion of foliar proteins [10], [37]. *PAL* is a key regulatory enzyme for the synthesis of phenolic compounds including SA. *PR1* mRNAs accumulate in response to increased SA levels and was utilized as a SA-responsive marker transcript. Changes in the levels of defense gene mRNAs were determined in fully expanded tomato leaves after *Lps* graft infection and in response to *Lps-*infected psyllids. Since *Lps*-free psyllids were not available and could not be cured of infection in this study (discussed below), it is not possible to directly assess gene expression changes to *Lps*-free psyllids. However, inoculation of tomato plants with *Lps* by grafting allowed the impact of *Lps* infection without its vector to be determined.

**Figure 1:**
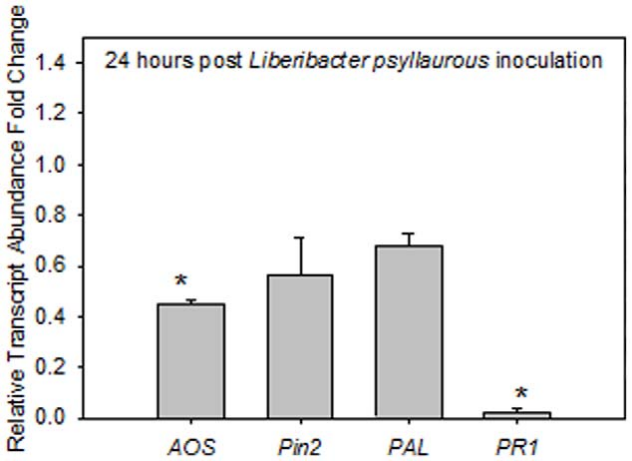
*Lps* down-regulates transcripts related to SA and JA signaling. Transcript abundance (mean ± SD) of four defense-response genes in fully expanded leaves from plants 24 hr after graft inoculation with *Lps*. *Allene oxide synthase* (*AOS*), *Proteinase inhibitor 2* (*Pin2*), *Phenylalanine ammonia-lyase 5* (*PAL5*) and *Pathogenesis-related 1* (*PR1*). Significant regulation from graft controls (dashed line = control transcript abundance) is indicated by an asterisk (*P*<0.05).

*Lps*-infected or *Lps-*free scions were grafted to *Lps*-free root stocks with three mature leaves. Graft transmission of *Lps* was rapid (Table 2). Within 24 hr after *Lps* inoculation via grafts, *Lps* was detected in the leaves of the grafted root stock (Table 2), whereas no *Lps* was detected in the plants using non-infected scions. Defense gene transcripts were measured in the root stock leaves from plants with *Lps*-infected and *Lps*-free (graft control) grafts. The graft control plants were used to account for any wound responses that occurred within the first 24 hr after grafting. Relative to the graft control plants, *AOS* and *PR1* mRNA levels declined (Fig. 1; *P*<0.05). While a decrease in *PAL* and *Pin2* transcripts were noted, it was not significantly different from controls (Fig. 1). Overall, *Lps* decreases transcript accumulation regulated by both JA- and SA- signaling.

**Table 2:** Number of plants infected by *Liberibacter psyllaurous* in psyllids and graft- inoculated tomato after 24 hr, 76 hr or 6 days.

	Number of *Lps*-positive plants^c^
Plant Material	24 hr	76 hr	6 days
*Lps* in the absence of vector^a^			
*Lps* inoculated	3	3	3
Graft Control	0	0	0
Vector-transmitted *Lps* b			
1^st^ and 2^nd^instar-infested leaves	3	3	3
3^rd^ instar-infested leaves	3	3	2
5^th^ instar- infested leaves	3	3	3
Adult-infested leaves	2	2	3
Uninfested control leaves	0	0	0

a Leaves from root stocks that were grafted to *Lps*-infected plants (*Lps* inoculated; N = 3) plants or uninfected scions (graft control; N = 3) were collected at 24 hr, 76 hr or 6 days after grafting.

b Leaves from tomato plants (N = 3) that were infested with 25 *B. cockerelli* instars or adults, were collected at 24 hr, 76 hr or 6 days after infestation.

c The presence of *Lps* in *B. cockerelli*-infested or *Lps*-graft inoculated tomato leaves was detected by PCR (see *Materials and Methods*).

### Tomato responses to *Lps* in association with its psyllid vector

**Figure 2:**
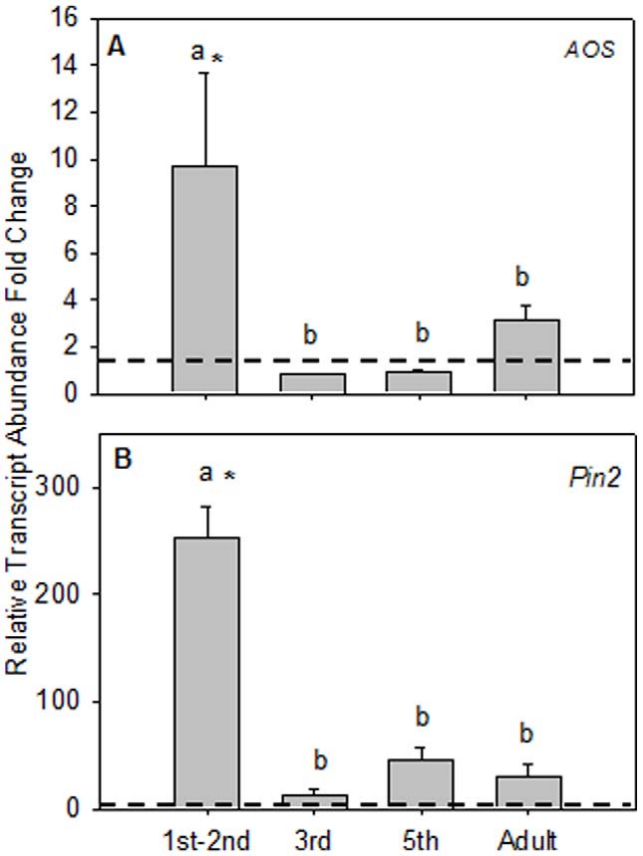
Young tomato psyllids induce jasmonic acid signaling transcripts. Relative transcript abundance (mean ± SE) of *AOS* and *Pin2* in fully expanded leaves exposed to 4 different developmental stages of tomato psyllid (1^st^–2^nd^, 3^rd^, 5^th^, and adult) for 24 hr. (A) *AOS*. (B) *Pin2*. Data is presented relative to uninfested control. Significantly different regulation from undamaged controls (dashed lines = control transcript abundance) is indicated by an asterisk and significant differences between insect developmental stages are indicated by letters (*P*<0.05).

To assess the changes in tomato defense gene expression after vector feeding, individual leaves of tomato plants were infested for 24 hrs with 25 infected psyllid adults or nymphs in their 1^st^–2^nd^, 3^rd^ or 5^th^ instars. First, in regard to JA signaling, *AOS* mRNA abundance was influenced by the developmental stage of *B. cockerelli* that was feeding on the plant (Fig. 2A; *P* = 0.0299); 1^st^–2^nd^ instar feeding caused a significant increase in *AOS* mRNA levels relative to uninfested control plants (Fig. 2A; *P*<0.05, Pairwise comparison LSD). In contrast, *AOS* transcripts were not significantly up-regulated after feeding by adults, 3^rd^ or 5^th^ instars relative to control plants (Fig. 2A; *P*>0.05, Pairwise comparison LSD).

Similar to *AOS*, the JA defense transcript *Pin2* was strongly influenced by the psyllid's developmental stage (Fig. 2B; *P*<0.0001). Feeding by 1^st^ instars caused a significant ∼250× increase in *Pin2* transcripts relative to uninfested leaves (Fig. 2B; *P*<0.05, Pairwise comparison LSD). In contrast, relative to 1^st^ instar feeding, *Pin2* mRNAs levels were more than ∼5 fold lower in the 3^rd^ and 5^th^ instars and adult treatments; *Pin2* transcript levels were not statistically different between 3^rd^, 5^th^ instars, and psyllid adults (Fig. 2B; *P*>0.05, Pairwise comparison LSD).

**Figure 3:**
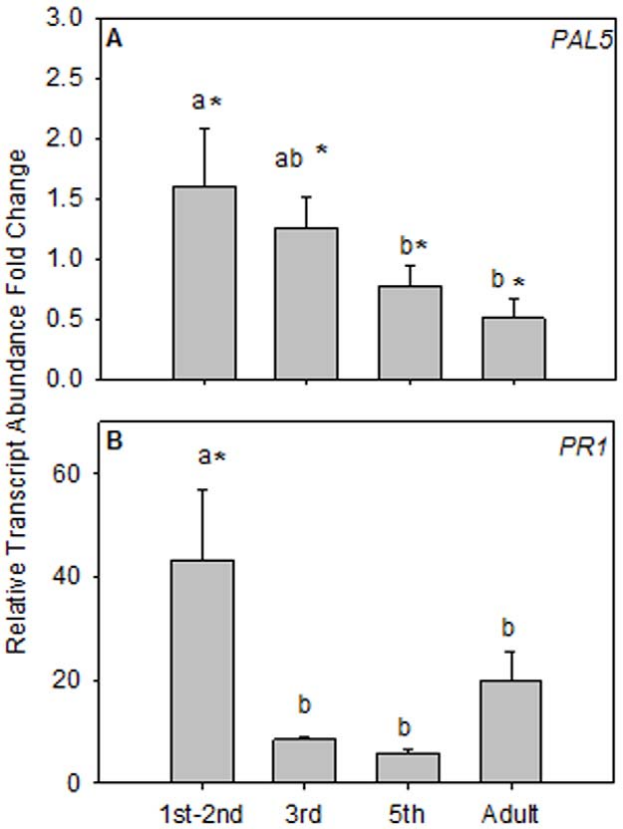
Young tomato psyllids induce salicylic acid regulated transcripts. Transcript abundance (mean ± SE) for *PAL5* and *PR1* in fully expanded leaves exposed to 4 different developmental stages of tomato psyllid (1^st^–2^nd^, 3^rd^, 5^th^, and adult) after 24 hr of insect feeding: (A) *PAL5*. (B) *PR1*. Significantly different regulation from undamaged controls (dashed lines = control transcript abundance) is indicated by an asterisk and significant differences between insect developmental stages are indicated by letters (*P*<0.05).

*PAL5*, a key biosynthesis enzyme in the SA pathway, is complexly regulated and its mRNAs accumulate in response to feeding by some hemipterans and the methyl form of JA (MeJA), and is negatively regulated by SA [16]. Psyllid developmental stage had a significant impact on *PAL5* transcript levels (Fig. 3A; *P* = 0.0015). Compared to uninfested leaves, there was an increase in *PAL5* mRNA levels after 1^st^–2^nd^ and 3^rd^ instar feeding (Fig. 3A; *P*<0.05, Pairwise comparison LSD). In contrast, 5^th^ instars and adult psyllids caused a significant reduction in *PAL5* transcript levels relative to undamaged controls and 1^st^ instar feeding (Fig. 3A; *P*<0.05, Pairwise comparison LSD).

*PR1* mRNAs are known to accumulate after biotic stress, SA and MeJA induction [16], [38], [39]. Abundance of *PR1* depended on the psyllid life stage feeding (Fig. 3B; *P* = 0.017). Within 24 hr of feeding, tomato plants infested with 1^st^–2^n^ instars accumulated the highest levels of *PR1* mRNA relative to other life stages of insect and undamaged controls (Fig. 3B; *P*<0.05, Pairwise comparison LSD).

### Efficiency of *Lps* transmission to tomato plants

Plants in 24 hour defense-response gene experiments were tested for *Lps* infection and disease symptoms. *Lps* transmission and disease symptoms were also observed after 72 hrs and 6 days of feeding to better characterize infection and disease symptom dynamics after 24 hrs of feeding for each lifestage. *Lps* was not detected in the uninfested control plants. *Lps* was successfully transmitted to tomato plants by all *Lps*-infected psyllid life stages (Chi-square = 3.889, df = 3, sig. = 0.274, Table 2). *Lps* was detected in most psyllid-infested plants after 24 hr, 72 hr, or 6 days of feeding (Table 2). The only insect life stage that did not transmit *Lps* at 100% efficiency was the adult (Table 2); adults transmitted *Lps* with an efficiency of 78%. In addition, the amount of time psyllids were allowed to feed on tomato plants and incubation time after graft inoculation (24 hr, 72 hr, and 6 days) had no significant effect on *Lps* infection success (Chi-square = 0.00, df = 2, sig. = 1.0; Table 2). Collectively these results indicate that *Lps* infection is rapid, occurring within 24 hr of infestation (Table 2). Infected and uninfected plants remained alive for over 72 days (the last day of observation) and *Lps*-infected plants developed qualitative psyllid yellows symptoms [8] within 3 weeks after infection relative to control plants.

### Infection and titer of *Lps* in *B. cockerelli* throughout development

Vertical transmission of *Lps* from infected gravid females to psyllid eggs was moderate to high and occurred in all six isofemale lines, ranging from 46.7–87.5% infection (Table 3). Since *Lps* is vertically transmitted from psyllid mother to egg at moderate to high frequencies (Table 3), we further assessed psyllid infection frequencies and titer throughout psyllid development for all psyllids used in 24 hr defense-response gene trials. For 72 hr and 6 day plant infection and symptom trials we also detected psyllid *Lps* infection frequency but not titer for different psyllid lifestages. For 24 hr defense-response gene trials *Lps* was detected in 96% of 1^st^–2^nd^ and 3^rd^ instar nymphs, 97.4% of adult psyllids, and 100% of 5^th^ instar psyllids (N = 75 individuals per instar). Only eight psyllids out of the 300 used in all trials did not have detectable levels of *Lps*. These insects included: one 1^st^–2^nd^ instar nymph from a 24-hr treatment, two 1^st^–2^nd^ instar nymphs in a 6-day treatment, one 3^rd^ instar nymph from a 24-hr, 72-hr, and 6-day treatment, and two adults from a 24-hr treatment. Consequently the majority of psyllid individuals used in transmission trials and the 24-hr plant transcript treatments were infected with *Lps*, and infection frequency among all psyllid individuals was very high regardless of psyllid life stage.

**Table 3:** Psyllid isofemale line egg infection frequencies of *Liberibacter psyllaurous*.

	Total Number of eggs^a^	Number of *Lps* infected eggs^b^	% Infection
Isofemale 1	8	7	87.5
Isofemale 2	4	3	75.0
Isofemale 3	19	11	57.9
Isofemale 4	14	11	78.6
Isofemale 5	15	7	46.7
Isofemale 6	11	6	54.5

a Psyllid egg extracts that were positive for the psyllid nuclear gene *FORKHEAD* (see *Materials and Methods*).

b *Lps* egg infection was determined by PCR using primer sets that detected two different bacterial gene intergenic regions (IGS and 50 S rRNA; see *Materials and Methods*).

**Figure 4:**
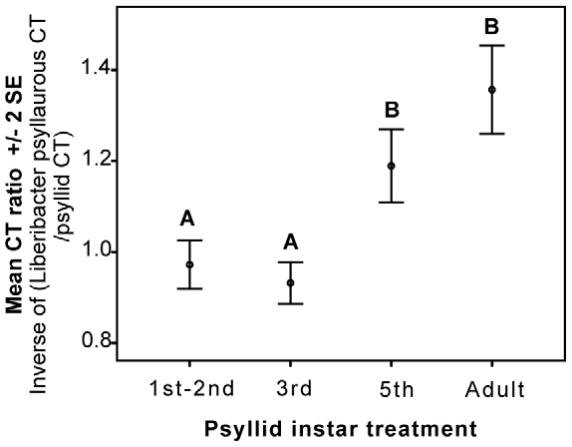
Young tomato psyllids carry the lowest titer of *Lps*. Relative abundance of the *Lps IGS* gene for four psyllid life stages compared to the single-copy insect host gene (*FORKHEAD*). Error bars are the 99% confidence interval of the mean. Letters indicate that treatments are significantly different at a 0.01 level using *FORKHEAD* as a covariate in ANCOVA. N = 24 psyllids per life stage.

Titer of *Lps* within psyllid individuals from the 24 hr defense-response trials depended on psyllid life stage; 5^th^ instar and adult psyllids had higher bacterium titers per insect cell relative to 1^st^, 2^nd^, and 3^rd^ instars (Fig. 4). There was a significant difference in *Lps* CT values among psyllid instars (F = 40.875, sig.<0.001, R^2^ = 0.627, corrected total d.f. = 95, Fig. 4). Scheffe post-hoc tests showed that *Lps* CT values of fifth instar nymphs were not significantly different from adult stage individuals (*P* = 0.226). Both fifth instar and adult stage treatments were significantly lower in CT values (i.e. higher in titer) relative to 1^st^ and 2^nd^ instar nymphs and 3^rd^ instar nymphs (*P*<0.01; Fig. 4). *Lps* CT ratios of 1^st^ and 2^nd^ instar nymphs were not significantly different from 3^rd^ instar nymphs (*P* = 1.00). Overall *Lps* titer increased as the psyllid aged.

### Anti-biotic treatment experiment of psyllids

In order to develop infected *Liberibacter psyllaurous* (*Lps*) and uninfected psyllid strains, techniques similar to those used to cure endosymbionts from aphids were used. The methods tested were ampicillin injection into host plants [40] and into gravid females [41]. Although these treatments were more tedious to conduct on sexual psyllids relative to clonal aphids, numerous attempts to cure *Lps* from psyllids using both techniques were unsuccessful.

When psyllids were reared on ampicillin-treated plants, *Lps* infection frequencies of 5^th^ instar psyllids initially declined to 50% but after three generations returned to the previous infection levels (100%). Microinjection of ampicillin into gravid psyllids caused high mortality of females relative to the buffer controls. Furthermore, the small numbers of females that survived the treatment and produced progeny were not cured of the *Lps* infection. The inability to recover *Lps*-free psyllids may be due to the fact that the psyllid line used in these studies may be codependent on *Lps* or that the antibiotic treatments, which are successful in curing endosymbionts from pea aphids, were less effective in psyllids.

## Discussion

Plant responses to the tomato psyllid and its endosymbiont-like pathogen *Lps* are complex and the data presented here suggest that *Lps* may influence the suite of plant defenses deployed upon psyllid infestation. When *Lps* is transmitted to tomatoes in the absence of its vector, there was a suppression of *PR1* and *AOS* mRNAs relative to graph control plants. These data suggest that *Lps* suppresses some plant defenses regulated by JA and SA. In contrast to graft-transmitted *Lps*, all four defense mRNAs (*PAL5*, *PR1*, *Pin2*, *AOS*) significantly increased during the early developmental stages (1^st^ and 2^nd^ instar) of infected psyllid feeding.(Figs. 1, 2, 3). When older infected developmental lifestages were examined (3^rd^ and 5^th^ instars and adult psyllids) striking differences in tomato expression were revealed relative to 1^st^ and 2^nd^ instar treatments. Compared to young psyllid feeding s SA and JA associated transcripts were suppressed after feeding by older psyllids, similar to infected graphing treatments (Figs. 1, 2, 3). The activation of plant defense-response genes was inversely correlated with *Lps* relative titers in the insect vector. Psyllids in their 1^st^, 2^nd^ and 3^rd^ instar supported smaller *Lps* populations relative to 5^th^ instars and adults (Fig. 4). The pattern of defense-response gene expression when *Lps* titer in psyllids was high emulated the response to *Lps* in isolation.

Collectively, these data indicate that tomatoes respond differentially to psyllids in different life stages. However, while well correlated, the titer of *Lps* in psyllids may only partially explain the life-stage specific variation in transcript abundance. This is suggested by the fact that *Lps* titers are low in 3^rd^ instar psyllids (Fig. 4), yet defense gene mRNAs accumulate to lower levels than after 1^st^ and 2^nd^ instar feeding (Fig. 2A,B; Fig. 3B). This lifestage variation may be attributed to how efficient a particular psyllid instar can vector *Lps* into its host plant, or the elicitors or effectors that are delivered in the insect saliva or released due to plant cell damage [11], [42]. Specific elicitors derived from developmental stage-specific feeding behaviors or insect secretions cannot be discounted based on our experimental design [43], [44]. For example, psyllid elicitors unique to 1^st^ and 2^nd^ instar nymphs could be produced in oral secretions and/or through differences in damage compared to more mature psyllids. Future purification and characterization of elicitors/effectors involved should shed light on this relationship and may potentially broaden the list of exogenous cues recognized by the plant surveillance mechanism that trigger plant defense.

This study showed that *Lps* caused a substantial reduction of JA/ET- and SA-regulated plant defense transcripts relative to uninfected controls. *Lps* may suppress plant defenses by introducing small molecules or protein effectors to promote virulence. Pathogen-derived effectors can antagonize plant immunity through a diversity of processes, including alterations in mRNA levels [45]–[48]. Many of these effectors are transmitted from microbes by a Type III secretion system [49]. But similar to other Alphaproteobacteria pathogens, *Liberibacter* species do not possess a Type III secretion system and their associated effectors [50], [51]. Nevertheless, *Lps* does possess a Type I secretion system and an exotoxin (a putative hemolysin protein; YP_004062617), which may be secreted by this system [51]. *Lps* also encodes for a FTR-1-like high affinity iron transporter that is associated with plant virulence in other plant pathogens [51]. It is clear that the mechanisms of *Lps* evasion of plant defenses warrant further investigation.

This study has demonstrated the complexity of the tomato-psyllid-*Lps* association and its probable impacts on psyllid success. Like other beneficial or deleterious heritable insect endosymbionts that are facultative and specific to a certain insect phyla [52], *Lps* occurs sporadically among individuals in field populations [53], [54] and is persistently associated with the tomato psyllid species. Nachappa et al. 2012 found that infected *Lps* tomato psyllids from the field displayed 1.6× lower fecundity and 1.5× lower nymphal survival on tomato relative to un-infected tomato psyllids collected from the field. Nevertheless two different psyllid nuclear and cytoplasmic genotypes were used during trials for infected versus un-infected comparisons and thus strain effects cannot be excluded as the major factors driving relative fitness effects.

*Lps* has an intimate and potentially long-term association with its insect host given the efficient vertical transmission of *Lps* to psyllid eggs (Table 3), increasing frequency of *Lps* infection throughout psyllid development [8], [53], increasing titer of *Lps* during the psyllid life cycle (Fig. 4), and the phylogenetic conservation of other *Liberibacter* species to the psyllid phylum. Furthermore, *Lps* in the absence of its vector and psyllids with higher *Lps* titers (3^rd^–5^th^ instars and adults) were able to suppress sentinel defense-response genes regulated by the JA- and SA-signaling pathways. This markedly contrasts to 1^st^ instars that have significantly lower *Lps* titers and strongly activate these sentinel defense response genes. Collectively, these data suggest that bacterial pathogens, like *Lps*, that are dependent on a specific insect vector may manipulate plant signaling and defensive responses to increase both their success and the success of their obligate insect vector on their host plant. In nature, mixed life stages of the tomato psyllid feed on a particular host plant, and consequently younger, less mobile instars may also benefit from this symbiotic interaction. Further studies using psyllids with and without symbionts on different host plants, and plants compromised in the different signaling pathways will determine whether or not *Lps* confers a selective advantage for psyllids.

## Materials and Methods

### Plant growth and insect rearing

*Solanum lycopersicum* L. cv. Moneymaker (Botanical Interests TM) seeds were planted in 473-mL pots with sterilized University of California (UC) soil mix three and fertilized with 1.25 mL of Osmocote Smart-Release flower and vegetable plant food fertilizer® (Scotts-Sierra Horticultural Products Company, Marysville, OH, USA) per plant. Plants were grown under greenhouse conditions (24–25°C) and metal halide grow lights (16 hr light∶8 hr dark) were used to supplement natural lighting. The plants used in all experiments detailed below were one month old and at the four-leaf stage at the start of trials. One day before psyllid infestations, plants were sorted into treatment and control groups. Plants were paired by leaf developmental stage among treatment and control groups.

*B. cockerelli* (Texas line, TX-Lipsy) colonies were maintained as described in Hansen et al. [8]. Psyllid adults in the TX-Lipsy line maintained >99% infection with *Lps* and were reared on tomato plants [8]. Instars were determined as described in Hansen et al. [8]. To further understand the association between *Lps* and the tomato psyllid, vertical transmission of the bacterium via psyllid eggs was examined in six isofemale lines (Table 3). For trials examining *Lps* infection in isofemale line eggs, six 10 day old mated females reared on infected potato were each isolated in a clipcage on their own tomato host plant. After 24 hrs, gravid females were removed from plants and eggs were allowed to develop for three days on the host plant before they were sampled. Before eggs were screened for *Lps* infection with PCR, the leaf and attached eggs were surface sterilized with 10% bleach (Clorox ®, Oakland, CA) for five minutes and then triple rinsed with sterilized double distilled water. Eggs were then removed from the leaf using heat sterilized insect pins under a dissecting microscope. Care was taken not too severe or puncture the leaf surface during this process.

### Whole plant infestations, RNA isolation, and microarray analysis

Because of limitations in technology we had at the time, we were not able to use more sensitive methods to examine global transcriptional profiles (for example RNAseq). However, trends were evident in results and we used these trends as an important justification for our sample design of the next round of trials using qPCR (next section).

Psyllid infestations of tomatoes were performed in a greenhouse (25–28°C, 16 hr light∶8 hr dark). All plants were encased individually in mesh bags. For half of the plants (N = 16), twenty-five adult psyllid females were released within the mesh bags encasing the plants (infested). The remaining group of plants in mesh bags (N = 16) received no psyllids (control plants, uninfested). After two days, the adults were removed from the infested plants and eggs were allowed to hatch. Eggs did not hatch synchronously, however time points were chosen when most psyllids in an infestation were at a particular life stage. At each time point, leaflets were collected from four different infested or uninfested plants and pooled. Plants were not used for more than one collection time point. Infested leaflets were harvested at 0 days (0-d control), 2 days (adult feeding/egg deposition), 10 days (1^st^–2^nd^ instar feeding), and 15 days (3^rd^–5^th^ instar feeding) after infestation. Developmentally matched leaves were harvested from uninfested control plants at 0, 2, 10, and 15 days and are referred to as the uninfested control reference (day 0) and the day 2, 10 and 15 uninfested controls. Harvested leaves were flash frozen in liquid nitrogen and stored at −80°C until use. The time-course experiment was executed 3 times.

Total RNA was extracted from each tissue sample according to the protocol recommended by TIGR (The Institute for Genomic Research; Rockville, MD). Briefly, leaves were ground in liquid nitrogen and total RNA was isolated using a hot phenol protocol (http://jcvi.org/potato/sol_ma_protocols.shtml). Total RNA was treated with DNase and further purified using the SV total RNA isolation kit (Promega, Madison, WI, USA). RNA concentrations were determined spectrophotometrically and integrity was verified on a 1.2% formaldehyde agarose gel [55].

For global transcriptional profiles, the TIGR potato microarray (10 K version 3) was used containing ∼10,000 annotated cDNA clones spotted as duplicates on the array. At the time the experiment was conducted (2004), cDNA microarrays were the best technology available for our budget. Detailed information about this microarray can be found at the TIGR web site (http://jcvi.org/potato/sol_ma_microarrays.shtml). A potato array was used because Solanaceous plant genomes are similar with respect to gene content, genome organization and nucleotide sequence conservation. For example, see the transcriptional analysis of several Solanaceous plants performed by Robin Buell [56].

All steps of microarray processing to obtain raw data (cDNA production, cDNA labeling, microarray hybridization, data quantification) were carried out by the TIGR Expression Profiling Service according to published methods (http://jcvi.org/potato/sol_ma_protocols.shtml). Briefly, hybridizations were conducted in a reference pool design. All uninfested (2, 10, 15 d) and infested (2, 10, 15 d) treatments (cDNA labeled with Cy3) were hybridized to slides with the reference (0 d) uninfested control pool (cDNA labeled with Cy5). Spot data was extracted using GENEPIX at TIGR (ver. 5.0 Pro: Axon Instruments, Union City, CA, USA). In compliance with MIAME standards our data output obtained from GENEPIX are publicly available and can be downloaded through a database maintained at the TIGR Web site (http://www.tigr.org/tigr-scripts/sgedb/studies_SGED.pl).

Using the LIMMA ([57]: http://bioinf.wehi.edu.au/limma/) package for the statistical software R, data sets were background corrected and normalized. Mean expression ratio from the duplicate spots per clone on each array were calculated prior to statistical analysis. To dissect true psyllid responsive transcripts from developmental changes in the plants, linear models were used with contrast matrixes, facilitating indirect comparisons between psyllid damaged tissue and developmentally matched controls. If the infested/reference ratio is greater than the control/reference ratio (a positive log2 (fold change)) value, a gene is designated as “up regulated”. Similarly if the infested/reference ratio is less than the control/reference ratio (a negative log2 (fold change)) value, a gene will be considered down-regulated.

Linear models were implemented and FDR-adjusted P values were calculated in LIMMA to identify genes that may be differentially expressed. Because expression levels were low and variation was high, there were no statistical differences in transcript expression profiles among treatments (Criteria: *P*<0.05 and a fold change of greater than 2) after psyllid infestation compared to developmentally matched controls. For further analysis the top 149 transcripts expressed in each treatment are available in Table S1, S2, and S3. The details of the experiment and raw microarray data are available and can be downloaded from the TIGR Solanaceae Gene Expression Database (ftp://ftp.tigr.org/pub/data/s_tuberosum/SGED/078_Paine/).

### Infestation of tomato with different life stages of *Lps*-infected psyllids

One-month-old tomato plants were challenged with *Lps*-infected psyllids of different developmental stages. For all infestations, 25 individual insects belonging to a particular instar were confined on a single leaflet per plant using a clip-cage [55] and allowed to feed for 24 hours. Four psyllid life stage inoculation treatments were evaluated: 1^st^ and 2^nd^ instar; 3^rd^ instar; 5^th^ instar; and adult. Three replicates were conducted for each insect developmental stage. Five healthy plants (psyllid and *Lps-*free) with clip cages were also included within trials as controls for psyllid infested plants. Each experiment replicate was isolated in an individual mesh insect cage (Bugdorm, MegaView Science Education Services Co., Ltd., Taichung, TAIWAN) to prevent other insects from contaminating plants during trials.

At the end of the infestation period, all psyllids and eggs were quickly removed from tomato leaves under a microscope before petiole and leaf midribs were cut from infested leaves (this is a crucial step since *Lps* is present in psyllid eggs [8]; Table 3). Leaf midribs and petioles were cut from leaf tissue using new sterilized razor blades, disposable Petri dishes, and disposable gloves for each sample to prevent sample contamination. Insects, leaf midribs/petioles and leaf blade were collected separately and flash frozen in liquid nitrogen and kept at −80°C until DNA and RNA extractions were performed. All psyllids used in trials were individually PCR screened for the presence of *Lps* after leaf samples were collected (see below).

### Vector-free transmission of *Lps* to tomato leaves

Numerous attempts to develop an uninfected psyllid line (*Lps*-free line) from a naturally infected TX-Lipsy line were unsuccessful. Insects were treated with ampicillin through host plants and by direct injection into gravid females according to Douglas and Prosser [40] and Koga et al. [41], respectively. Infection came back after the first treatment and in the second there was high mortality of females and progeny from surviving females were not cured of the infection. Therefore, the impact of *Lps* on tomato gene expression had to be assessed in the absence of its vector.

Tomato plants infected with *Lps* were generated by grafting a *Lps*-infected scion to a non-infected tomato root stock. Control plants were constructed by grafting a non-infected scion to a non-infected root stock. All plants used in this experiment were one month old. *Lps*-infected scions were isolated from plants infested with psyllids for 2 weeks. Infected tomato plants were stripped of all leaves with a clean razor blade, triple rinsed with water to remove all psyllids, and sprayed with 2% Sunspray ultra-fine oil (Sunoco Inc., Aartselaar, Belgium) to kill any small nymphs and eggs potentially remaining on infected scions. Infected shoots were immediately tongue grafted to uninfected tomato plants (the root stock) 24 hours after insect removal; three whorls of leaves remained on the root stock. To control for the wounding that occurs during grafting, uninfected tomato shoots were grafted to the root stock (grafting control) as described above. Grafting treatments and controls were performed at the same time as psyllid life-stage treatments in an insect rearing room at 25+/−1°C with a photoperiod of 14∶10 (L∶D) using metal halide grow lights. Grafting experiments were isolated in a separate mesh cage to protect plants from insect infestations.

Leaves from the root stocks of the *Lps*-infected scion plants (N = 5) or uninfected scion plants (N = 5) were collected at 24 hr, 72 hr, and 6 days after grafting to insure successful *Lps* infection. *Lps* presence in root stock leaves was determined with PCR (see below). Because *Lps* was detected in leaves of root stocks 24 hours after grafting to infected scions, RNAs were isolated and defense gene mRNA levels were determined for the 24-hr samples. The experiments were repeated three times.

### DNA and RNA isolation

DNA was extracted as described in Hansen et al. [8] from insects, eggs and plant midribs/petioles collected in the insect life stage and *Lps* graft experiment above. Bleach sterilized and autoclaved mortar and pestles were used to grind plant tissue in liquid nitrogen before plant extractions were performed, and 100 mg of ground leaf midrib/petioles were used for each extraction.

Total RNA was extracted from the 24-hour psyllid-infested and control leaves using a guanidinium thiocyanate-acid phenol based method [56]. To clean up the RNA, a modified cetyl trimethylammonium bromide (CTAB) method was used [58]. Briefly, total RNA was extracted in 500 µL of hot CTAB extraction buffer. Samples were then incubated at 65°C for 10 minutes, centrifuged and the supernatant was removed. The supernatant was re-extracted with 500 µL of hot 5% CTAB solution for an additional 20 minutes. Following centrifugation, the supernatant was removed and extracted with chloroform-isoamyl alcohol (24∶1 [v/v]) three times and precipitated overnight with 10 M LiCl. RNA integrity was verified on a 1.2% formaldehyde agarose gel [55] and with a microfluidic visualization tool (Bioanalyzer, Agilent Technologies, Santa Clara CA,USA, http://www.algilent.com) at UCR's Genomics Core.

### PCR detection of *Lps* in the plant, insect and eggs

For *Lps* detection using PCR, two primer sets were used for two different *Lps* gene regions. The ribosomal intergenic space (IGS) was detected using 1611F and 480R primers [8] and 50 S rRNA region was detected using Bop-F and Bop-R primers (Table 4). To prevent pre-PCR carry-over contamination, a high fidelity polymerase (Fast Start High Fidelity PCR system, Roche, Indianapolis, IN, USA) with the ability to incorporate dUTP (Fermentas) was used for 50 S rRNA primers. This gene region has never been amplified before with dTTPs in contrast with the IGS primer set in our lab [59].

**Table 4:** Primer sets used in this study.

Primer Name	Sequence	Method
Bop-F	5′-CTCTAAGATTTCGGTTGGTT-3′	PCR
Bop-R	5′-TATATCTATCGTTGCACCAG-3′	PCR
Y DRAG Q-PCR- IGS-7F	5′-ATAGCTCAGGCGGTTAGAGTG–3′	qPCR
Y DRAG Q-PCR- IGS-7R	5′-CCTTGCCTGATTGAATGGTG-3′	qPCR
QPCR-FKhead_90F	5′-TGGACCTGTTCCCGTTCTAC-3′	qPCR
QPCR-Fkhead_188R	5′-TGCGAGGAACTTTCACAAAA-3′	qPCR
tomPAL-C-F	5′-CAATGGCTTGGACCTCAGAT-3′	qRT-PCR
tomPAL-C-R	5′-CCACCATGTAAGGCCTTGTT-3′	qRT-PCR
tomAOS-F	5′-TCTCTTCCTCTTCCTTCTCTTCACC-3′	qRT-PCR
tomAOS-R	5′-CGCCGGGTATAGTCCTGGTAGATA-3′	qRT-PCR
tomPR1-F	5′-CCGTGCAATTGTGGGTGTC-3′	qRT-PCR
tomPR1-R	5′-GAGTTGCGCCAGACTACTTGAGT-3′	qRT-PCR
tomPin2-F	5′-AATTATCCATCATGGCTGTTCAC-3′	qRT-PCR
tomPin2-R	5*′-*CCTTTTTGGATCAGATTCTCCTT-3′	qRT-PCR
tom18 s-F	5′-GAAACGGCTACCAATCCAAG-3′	qRT-PCR
tom18 s-R	5′-CCCCGTGTTAGGATTGGGT-3′	qRT-PCR

For both primer sets, PCR was performed in 25-µL reactions containing 1 µL of DNA template (concentration not determined), 0.2 mM each of dATP, dCTP, and dGTP, 0.4 mM of dTTP mix, 0.2 µM each primer, 1× PCR MgCl_2_-free buffer (Roche, Indianapolis, IN, USA), 5 mM MgCl_2_, 1 U polymerase (Roche, Indianapolis, IN, USA), and 1 U UDG when 50 s rRNA primers were used. For 1611F and 480R primers, amplifications were performed in a Mastercycler 5331 (Eppendorf, Hamburg, Germany) programmed as: an initial denaturing step of 95°C for 5 min; followed by 38 cycles of 95°C for 30 sec, 60°C for 50 sec, and 72°C for 1.5 min; and a final extension step of 72°C for 10 min. Thermocycler conditions for 50 s rRNA Bop-F and Bop-R primers (using UDG) were: an initial incubation of 37°C for 10 min, an initial denaturing/UDG deactivation step of 95°C for 10 min; followed by 38 cycles of 94°C for 30 sec, 60°C for 45 sec, and 72°C for 45 sec; and a final extension step of 72°C for 5 min. After PCR with 50 S rRNA primers, 0.1 U of uracil glycosylase inhibitor (New England Biolabs, Ipswich, MA, USA) was added to each PCR reaction. The PCR product was then incubated for 30 min at 37°C. Extraction and PCR negative controls and a PCR positive control were included in each assay.

Amplified DNA was visualized after electrophoresis on a 1% agarose gel stained with ethidium bromide (run at ∼4.9 V/cm for at least 1 hr). Amplified *Lps* DNA from each primer set (one positive from each plant treatment, one positive from an egg from each isofemale line, and one positive from psyllids used during trials) were cleaned using the Wizard® PCR Preps DNA Purification System (Promega, Madison, WI, USA) and direct sequenced in both directions at the UC Riverside Genomics Institute Core Instrumentation Facility using an Applied BioSystems 3730 DNA Analyzer with a Big-Dye® V3.1 kit (Applied BioSystems, Foster City, CA, USA). The Kruskal-Wallis test with the Chi-squared statistic was used to determine if there was a difference in *Lps* infection (presence/absence) in plant DNA extracts for time since inoculation and psyllid life stage treatments.

Insect DNA extracts positive for *Lps* presence were used to further quantify *Lps* titer within psyllids using quantitative PCR (qPCR) using a Rotor-Gene 3000 (Corbett Research, Rotor-gene 6.1) with a 72-well rotor was used for psyllid qPCR reactions. For qPCR, primers used for *Lps* quantification, which were developed in this study are: Y DRAG Q-PCR- IGS-7F and Y DRAG Q-PCR- IGS-7R (Table 4). DyNAmo HS, Sybr Green (Finnzymes, Lafayette, CO, USA) master mix, 0.9 mM of primer, 1 U of UDG, and 1 U of FastStart High Fidelity PCR System polymerase were all incorporated into a final volume of 18 µL master mix and 2 µL of psyllid DNA template extracted from individual psyllids. A single-copy psyllid reference gene (*FORKHEAD*) was also amplified with quantitative PCR to control for body mass variations between psyllid lifestages (QPCR-FKhead_90F and QPCR-Fkhead_188R; Table 4). Reaction conditions for qPCR were an initial incubation of 50°C for 2 min, an initial denaturing/UDG deactivation step of 95°C for 15 min; followed by 40 cycles of 94°C for 10 sec, 60°C for 20 sec, and 72°C for 30 sec; and a final melting step of 72–95°C with a 1°C increase every 5 sec after an initial 45 sec 1°C increase.

Relative quantification of bacterial titer was modified from Bustin [59] using LinRegPCR 11.1 for data [60], [61]. After CT values were calculated for each qPCR technical replicate (four per sample) values were averaged per sample. To determine if there is a difference in *Lps* titer (CT, see above) between psyllid life stages an ANCOVA was used with *FORKHEAD* CT included as a covariate (to control for differences in psyllid biomass across life stage treatments). Post-hoc multiple comparison analyses of CT values were conducted between psyllid life stages using Scheffe's multiple comparisons. Parametric data were tested for normality using the Kolmogorov-Smirnov test. Type I error for all analyses is α = 0.01. All statistical analyses were conducted using SPSS Inc. 14.0 software.

### Detection of tomato transcripts using quantitative reverse transcriptase-PCR (qRT-PCR)

Expression of transcripts associated with signaling and defensive tomato genes were quantified for both psyllid infested and grafting treatments, to tease apart the influence of *Lps* on the plant with and without its insect host. *AOS* (*Allene oxide synthase*), *Pin2* (*Proteinase inhibitor 2*), *PAL5* (*Phenylalanine ammonia-lyase* 5), *PR1* (*Pathogenesis-related 1*; *P6*) and *HSP90* (to confirm microarray data) mRNA levels were determined using quantitative reverse transcriptase-PCR (qRT-PCR). Three internal standard primer sets were evaluated for highest efficiency and lowest variation across samples; *18 S rRNA was* used in experiments based on this criteria. Primers used for qRT-PCR were designed using Primer-Blast (http://www.ncbi.nlm.nih.gov/tools/primer-blast/index.cgi?LINK_LOC=NcbiHomeAd).

Total RNA (1 µg) was DNase (Rq1, Promega, Madison, WI, USA) treated to clean up RNA and used as the starting material for the qRT-PCR experiments. The first-strand cDNA synthesis was carried out with the ‘SuperScript’ kit (Invitrogen Technologies, Carlsbad, CA, USA) using oligo-dT as primer and recommended methods in kit. Reactions were carried out using 5 µL of the ‘Sybr green PCR master mix’ (Applied Biosystems, Carlsbad, CA, USA), with 800 nM of primer, in the 7500 instrument (Applied Biosystems, Carlsbad, CA, USA). The *PAL5*, *AOS*, *Pin2*, *PR1*, and *18 S* primers used for qRT-PCR are listed in Table 4.

The PCR reaction was initiated with incubation to 95°C for 10 min to activate the enzyme. Then, the following cycle was repeated 40 times: 95°C for 15 sec, 60°C for 15 sec, and 72°C for 15 sec. Three technical replicates were performed for each individual plant RNA sample, and a digital pipette was used for all qRT-PCR reactions. The raw CT values were averaged, quantified and analyzed according to the standard curve method (Applied Biosystems, Carlsbad, CA, USA), resulting in relative fold change. Psyllid-infested plant mRNA levels were expressed relative to undamaged controls with empty cages and *Lps-*graft-inoculated plant mRNA levels were expressed relative to graft-control plants.

Analysis of variance (ANOVA) was used to determine if there was a difference in transcript abundance in tomatoes infested with *Lps*-infected psyllids and infected with vector-free *Lps*. Post-hoc multiple comparison analyses of relative fold change values were conducted between psyllid-infested plants and *Lps*-inoculated plants using Least Significant Difference (LSD) Tests. All statistical analyses were conducted using SAS, version 9.

## Supporting Information

Table S1 The top 149 tomato transcripts expressed after tomato psyllid1^st^–2^nd^ instar feeding.
(XLS)

Table S2 The top 149 tomato transcripts expressed after tomato psyllid 3^rd^–5^th^ instar feeding.
(XLS)

Table S3 The top 149 tomato transcripts expressed after tomato psyllid adult feeding.
(XLS)

## References

[1] Purcell AH (1982). Insect vector relationships with prokaryotic plant pathogens.. Annual Review of Phytopathology.

[2] Davis MJ, Ying Z, Brunner BR, Pantoja, Ferwerda FH, Starr MP (1981). Fastidious bacteria of plant vascular tissue and invertebrates (including so-called *Rickettsia*-like bacteria).. Selections from the Prokaryotes: Phytopathogenic Bacteria.

[3] Davis MJ, Ying ZT, Brunner BR, Pantoja A, Ferwerda FH (1998). Rickettsial relative associated with papaya bunchy top disease.. Current Microbiology.

[4] Frosch M (1983). Occurrence and distribution of the agent of the latent rosette (witches broom) disease of sugar beet (*Beta vulgaris*) in its insect vector *Piesma quadratum* Fieb (Heteroptera, Piesmidae) Zeitschrift Fur Angewandte.. Entomologie-Journal of Applied Entomology.

[5] Gatineau F, Jacob N, Vautrin S, Larrue J, Lherminier J (2002). Association with the syndrome “basses richesses” of sugar beet of a phytoplasma and a bacterium-like organism transmitted by a Pentastiridius sp.. Phytopathology.

[6] Semetey O, Bressan A, Richard-Molard M, Boudon-Padieu E (2007). Monitoring of proteobacteria and phytoplasma in sugar beets naturally or experimentally affected by the disease syndrome ‘Basses richesses’.. European Journal of Plant Pathology.

[7] van den Berg MA, van Vuuren SP, Deacon VE (1992). Studies on the greening disease transmission by the citrus psylla,*Trioza erytreae* (Hemiptera:Triozidae).. Israel Journal of Enomology.

[8] Hansen AK, Trumble JT, Stouthamer R, Paine TD (2008). A new huanglongbing species, “*Candidatus liberibacter psyllaurous*,” found to infect tomato and potato, is vectored by the psyllid *Bactericera cockerelli* (Sulc).. Applied and Environmental Microbiology.

[9] Glazebrook J (2005). Contrasting mechanisms of defense against biotrophic and necrotrophic pathogens.. Annual Review of Phytopathology.

[10] Howe GA, Jander G (2008). Plant immunity to insect herbivores.. Annual Review of Plant Biology.

[11] Walling LL (2009). Adaptive defense responses to pathogens and pests.. Advances in Botanical Research.

[12] McSteen P, Zhao Y (2008). Plant hormones and signaling: Common themes and new developments.. Developmental Cell.

[13] Stout MJ, Thaler JS, Thomma B (2006). Plant-mediated interactions between pathogenic microorganisms and herbivorous arthropods.. Annual Review of Entomology.

[14] Bari R, Jones J (2009). Role of plant hormones in plant defence responses.. Plant Molecular Biology.

[15] Kempema LA, Cui X, Holzer FM, Walling LL (2007). Arabidopsis transcriptome changes in response to phloem-feeding silverleaf whitefly nymphs. Similarities and distinctions in responses to aphids.. Plant Physiol.

[16] Puthoff DP, Holzer FM, Perring TM, Walling LL (2010). Tomato pathogenesis-related protein genes are expressed in response to *Trialeurodes vaporiorium* and *Bemisia tabaci* type B feeding.. Journal of Chemical Ecology.

[17] Kusnierczyk A, Winge P, Jorstad TS, Troczynska J, Rossiter JT (2008). Towards global understanding of plant defence against aphids - timing and dynamics of early Arabidopsis defence responses to cabbage aphid (*Brevicoryne brassicae*) attack.. Plant Cell and Environment.

[18] Zarate SI, Kempema LA, Walling LL (2007). Silverleaf whitefly induces salicylic acid defenses and suppresses effectual jasmonic acid defenses.. Plant Physiol.

[19] Mur LAJ, Kenton P, Atzorn R, Miersch O, Wasternack C (2006). The outcomes of concentration-specific interactions between salicylate and jasmonate signaling include synergy, antagonism, and oxidative stress leading to cell death.. Plant Physiology.

[20] Leon-Reyes A, Van der Does D, De Lange ES, Delker C, Wasternack C (2010). Salicylate-mediated suppression of jasmonate-responsive gene expression in Arabidopsis is targeted downstream of the jasmonate biosynthesis pathway.. Planta (Berlin).

[21] Verhage A, van Wees SCM, Pieterse CMJ (2010). Plant immunity: It's the hormones talking, but what do they say?. Plant Physiology.

[22] Gao LL, Anderson JP, Klingler JP, Nair RM, Edwards OR (2007). Involvement of the octadecanoid pathway in bluegreen aphid resistance in *Medicago truncatula*.. Molecular Plant-Microbe Interactions.

[23] Pieterse CMJ, Dicke M (2007). Plant interactions with microbes and insects: from molecular mechanisms to ecology.. Trends in Plant Science.

[24] Zhang PJ, Zheng SJ, van Loon JJA, Boland W, David A (2009). Whiteflies interfere with indirect plant defense against spider mites in Lima bean.. Proceedings of the National Academy of Sciences of the United States of America.

[25] Thaler JS, Agrawal AA, Halitschke R (2010). Salicylate-mediated interactions between pathogens and herbivores.. Ecology.

[26] Bhattarai KK, Xie QG, Pourshalimi D, Younglove T, Kaloshian I (2007). Coi1-dependent signaling pathway is not required for Mi-1-mediated potato aphid resistance.. Molecular Plant-Microbe Interactions.

[27] Bhattarai KK, Hagop SA, Kaloshian I, Eulgem T (2010). WRKY72-type transcription factors contribute to basal immunity in tomato and Arabidopsis as well as gene-for-gene resistance mediated by the tomato *R* gene *Mi-1*.. Plant Journal.

[28] Liefting LW, Sutherland PW, Ward LI, Paice KL, Weir BS (2009). A New ‘Candidatus Liberibacter’ Species Associated with Diseases of Solanaceous Crops.. Plant Disease.

[29] Knowlton GF, Thomas WL (1934). Host plants of the potato psyllid.. Journal of Economic Entomology.

[30] List GM (1939). The potato and tomato pysllid and its control on tomatoes.. Colorado Agricultural Experiment Station Bulletin.

[31] Pletsch DJ (1947). The potato psyllid *Paratrioza cokerelli* (Sulc.), its biology and control.. Montana Agricultural Experiment Station Bulletin.

[32] Wallis RL (1955). Ecological studies on the potato psyllid as a pest of potatoes.. USDA Technical Bulletin.

[33] Carter RD (1950).

[34] Rensink WA, Lee Y, Liu J, Iobst S, Ouyang S (2005). Comparative analyses of six solanaceous transcriptomes reveal a high degree of sequence conservation and species-specific transcripts.. BMC Genomics.

[35] Broekgaarden C, Poelman EH, Steenhuis G, Voorrips RE, Dicke M (2008). Responses of *Brassica oleracea* cultivars to infestation by the aphid *Brevicoryne brassicae*: an ecological and molecular approach.. Plant Cell and Environment.

[36] Delp G, Gradin T, Ahman I, Jonsson LMV (2009). Microarray analysis of the interaction between the aphid Rhopalosiphum padi and host plants reveals both differences and similarities between susceptible and partially resistant barley lines.. Molecular Genetics and Genomics.

[37] Howe GA (2004). Jasmonates as signals in the wound response.. Journal of Plant Growth Regulation.

[38] Chao WS, Gu Y-Q, Pautot V, Bray EA, Walling LL (1999). Leucine aminopeptidase RNAs, proteins and activities increase in response to water deficit, salinity and the wound signals - systemin, methyl jasmonate, and abscisic acid.. Plant Physiology (Rockville).

[39] de Ilarduya OM, Xie QG, Kaloshian I (2003). Aphid-induced defense responses in *Mi-1*-mediated compatible and incompatible tomato interactions.. Molecular Plant-Microbe Interactions.

[40] Douglas AE, Prosser WA (1992). Synthesis of the essential amino acid tryptophan in the pea aphid (*Acyrthosipon pisum*) symbiosis.. Journal of Insect Physiology.

[41] Koga R, Tsuchida T, Fukatsu T (2003). Changing partners in an obligate symbiosis: a facultative endosymbiont can compensate for loss of the essential endosymbiont *Buchnera* in an aphid.. Proceedings of the Royal Society of London Series B-Biological Sciences.

[42] Felton GW, Tumlinson JH (2008). Plant-insect dialogs: complex interactions at the plant-insect interface.. Current Opinion in Plant Biology.

[43] Takabayashi J, Takahashi S, Dicke M, Posthumus MA (1995). Developmental stage of herbivore *Pseudaletia separata* affects production of herbivore-induced synomone by corn plants.. Journal of Chemical Ecology.

[44] Tang JY, Zielinski RE, Zangerl AR, Crofts AR, Berenbaum MR (2006). The differential effects of herbivory by first and fourth instars of *Trichoplusia ni* (Lepidoptera ∶ Noctuidae) on photosynthesis in *Arabidopsis thaliana*.. Journal of Experimental Botany.

[45] Abramovitch RB, Anderson JC, Martin GB (2006). Bacterial elicitation and evasion of plant innate immunity.. Nature Reviews Molecular Cell Biology.

[46] Boller T, He SY (2009). Innate immunity in plants: An arms race between pattern recognition receptors in plants and effectors in microbial pathogens.. Science.

[47] Chisholm ST, Coaker G, Day B, Staskawicz BJ (2006). Host-microbe interactions: Shaping the evolution of the plant immune response.. Cell.

[48] Grant SR, Fisher EJ, Chang JH, Mole BM, Dangl JL (2006). Subterfuge and manipulation: Type III effector proteins of phytopathogenic bacteria.. Annual Review of Microbiology.

[49] Galán JE, Wolf-Watz H (2006). Protein delivery into eukaryotic cells by type III secretion machines.. Nature.

[50] Duan YP, Zhou LJ, Hall DG, Li WB, Doddapaneni H (2009). Complete genome sequence of citrus huanglongbing bacterium, ‘*Candidatus Liberibacter asiaticus*’ obtained through metagenomics.. Molecular Plant-Microbe Interactions.

[51] Lin H, Lou BH, Glynn JM, Doddapaneni H, Civerolo EL (2011). The complete genome sequence of ‘*Candidatus Liberibacter solanacearum*’, the bacterium associated with potato zebra chip disease.. Plos One.

[52] Oliver KM, Degnan PH, Burke GR, Moran NA (2010). Facultative symbionts in aphids and the horizontal transfer of ecologically important traits.. Annual Review of Entomology.

[53] Nachappa P, Levy J, Pierson E, Tamborindeguy C (2011). Diversity of endosymbionts in the potato psyllid, Bactericera cockerelli (Hemiptera: Triozidae) vector of zebra chip disease.. Curr Microbiol.

[54] Nachappa P, Shapiro AA, Tamborindeguy C (2012). Effect of ‘Candidatus Liberibacter solanacearum’ on Fitness of Its Insect Vector, Bactericera cockerelli (Hemiptera: Triozidae), on Tomato.. Phytopathology.

[55] Sambrook J, Fritsch EF, Maniatis T (1989). Molecular Cloning: A Laboratory Manual.

[56] Rensink W, Lee Y, Liu J, Iobst S, Ouyang S (2005). Comparative analyses of six solanaceous transcriptomes reveal a high degree of sequence conservation and species-specific transcripts.. BMC Genomics.

[57] Wettenhall JM, Smyth GK (2004). limmaGUI: A graphical user interface for linear modeling of microarray data.. Bioinformatics.

[58] Chang S, Puryear J, Cairney J (1993). A simple and efficient method for isolating RNA from pine trees.. Plant Molecular Biology Reports.

[59] Hartley JL, Rashtchian A (1993). Dealing with contamination: Enzymatic control of carryover contamination in PCR.. PCR Methods and Applications.

[60] Ramakers C, Ruijter JM, Deprez RHL, Moorman AFM (2003). Assumption-free analysis of quantitative real-time polymerase chain reaction (PCR) data.. Neuroscience Letters.

[61] Ruijter JM, Ramakers C, Hoogaars WMH, Karlen Y, Bakker O (2009). Amplification efficiency: linking baseline and bias in the analysis of quantitative PCR data.. Nucleic Acids Research.

