# Correction: Manipulation of Plant Defense Responses by the Tomato Psyllid (*Bactericerca cockerelli*) and Its Associated Endosymbiont *Candidatus* Liberibacter Psyllaurous

**DOI:** 10.1371/annotation/9903158b-c45c-44b9-b152-7ffb5bec0c32

**Published:** 2012-06-28

**Authors:** Clare L. Casteel, Allison K. Hansen, Linda L. Walling, Timothy D. Paine

Figure 1 and Figure 3 are incorrect. The correct Figure 1 and Figure 3 can be seen here:

Figure 1: 

**Figure:**
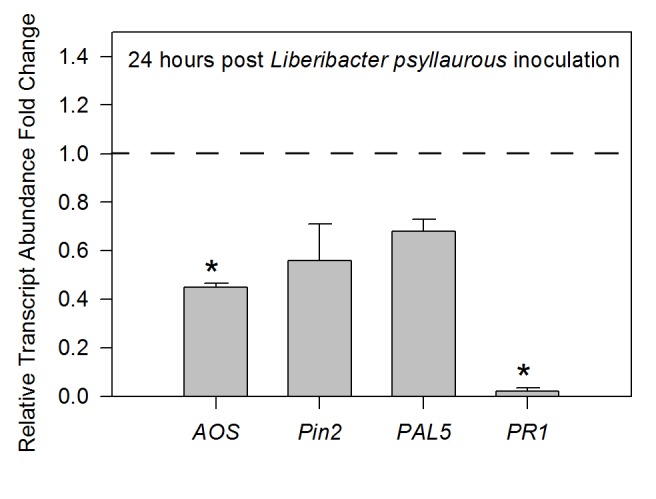


[^]

Figure 3: 

**Figure:**
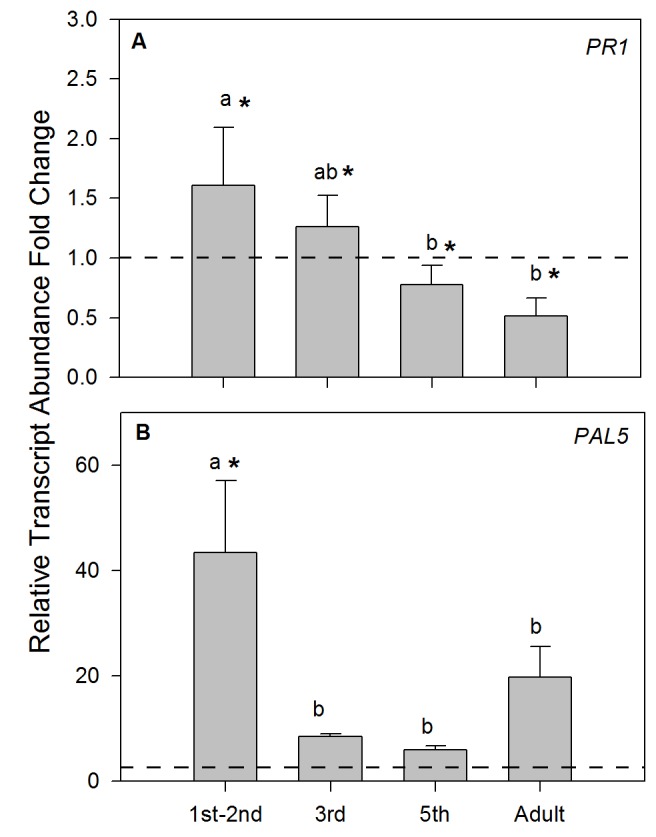


[^] 

